# Shear Stress as a Driver of Retinal Müller Glia Survival and Fibrotic Reprogramming

**DOI:** 10.1002/cbf.70224

**Published:** 2026-05-11

**Authors:** Laura Prieto‐López, Christian van Oterendorp, Elena Vecino, Xandra Pereiro

**Affiliations:** ^1^ Department of Cell Biology and Histology, Experimental Ophthalmo‐Biology Group University of Basque Country UPV/EHU Leioa Spain; ^2^ Department of Ophthalmology University Medical Center Göttingen Germany; ^3^ Begiker‐Ophthalmology Research Group BioBizkaia Health Research Institute Barakaldo Spain

**Keywords:** extracellular matrix, flow, Müller glia, shear stress, TGF‐β1, TRPV4

## Abstract

The retina is highly influenced by mechanical cues such as intraocular fluid movement and blood flow, generating shear stress implicated in both retinal development and pathology. Müller glia, as retinal mechanosensors, are uniquely positioned to respond to such forces. This study examined Müller glia responses to flow‐induced shear stress. Primary adult rat Müller glia were cultured in a single‐channel microfluidic system and exposed to fluid shear stress (10^−3^ dyn/cm^2^) for 24 h. Müller glia survival, morphology, and extracellular matrix (ECM) remodelling were evaluated. Expression of the mechanosensitive ion channel TRPV4, phosphorylated focal adhesion kinase (pFAK), and the pro‐fibrotic cytokine TGF‐β1 was analysed. TRPV4 and TGF‐β1 were pharmacologically inhibited to assess their functional roles. The results showed that shear stress enhanced Müller glia survival and reduced cell area. TRPV4 expression increased under flow, and its inhibition decreased survival and reversed morphological changes. Shear stress also elevated pFAK levels in a TRPV4‐dependent manner. Similarly, TGF‐β1 expression increased with flow, and its inhibition decreased survival and altered cell morphology under both static and flow conditions. ECM remodelling involved increased intracellular collagen I and IV levels and enhanced fibronectin deposition, both regulated by TGF‐β1. In conclusion, shear stress induces Müller glia survival, cytoskeletal remodelling, and selective ECM regulation via TRPV4 and TGF‐β1. TRPV4 acts upstream through pFAK signalling, while TGF‐β1 controls ECM remodelling. Together, these pathways initiate early remodelling and contraction, potentially driving retinal fibrosis post‐injury. Targeting TRPV4 and TGF‐β1 may offer therapeutic strategies to limit glial scarring and preserve retinal structure.

## Summary

- This study demonstrates that flow‐induced shear stress triggers adaptive and pro‐fibrotic responses in Müller glia through coordinated activation of TRPV4 and TGF‐β1 signalling pathways.
- Mechanical stimulation enhanced cell survival, promoted cytoskeletal and morphological remodelling, and increased pFAK activation in a TRPV4‐dependent manner, highlighting the role of mechanotransduction in retinal glial responses.
- In parallel, shear stress induced TGF‐β1 expression and selective extracellular matrix remodelling characterised by increased collagen I and IV production and fibronectin deposition.
- Together, these findings suggest that Müller glia convert retinal mechanical cues into early fibrotic remodelling processes, identifying TRPV4 and TGF‐β1 as promising therapeutic targets to limit glial scarring and preserve retinal integrity following injury.

## 1 Introduction

Mechanical forces within neural tissues are increasingly recognised as critical modulators of cell behaviour, influencing development, homoeostasis and pathology [1]. In the retina, a complex, stratified neural tissue, mechanical cues arise from both intrinsic properties, such as extracellular matrix (ECM) composition [2], and extrinsic influences like intraocular fluid dynamics and vitreoretinal interactions [3].

Müller glia, which extend radially across the retina and physically interact with neurons, blood vessels and ECM, are uniquely positioned to act as mechanosensors and responders [4, 5]. Indeed, Müller glia express different mechanoreceptors such as Transient Receptor Potential Vanilloid 4 (TRPV4), which facilitates calcium influx in response to mechanical stimuli [6]. Activation of TRPV4 in Müller glia has been linked to gliosis, cytokine release and neuroinflammatory signalling [7], triggering different cellular responses [8]. A key downstream effector of TRPV4 is the profibrotic cytokine transforming growth factor‐β1 (TGF‐β1), which promotes ECM synthesis and matrix stiffening [9]. This establishes a feedback loop that sustains Müller glia activation and exacerbates fibrosis. Supporting this, our group recently reported that Müller glia alter their phenotype and ECM deposition when exposed to varying substrate stiffness and high‐pressure conditions [10].

Beyond substrate stiffness and pressure, other mechanical forces, such as shear stress, play important roles in retinal homoeostasis. Shear stress (a tangential force exerted parallel to a surface) primarily arises in the retina from blood flow within the blood‐retina barrier (BRB). During angiogenesis, shear stress influences cellular orientation and vascular remodelling [11], and in adult tissues, it affects BRB integrity by regulating tight junction components [12]. Additionally, microfluidic systems exploiting shear stress have been employed to guide retinal ganglion cell axon regeneration in neurodegenerative models [13, 14, 15]. However, shear stress is also involved in various retinopathologies. Abnormal shear forces at the vitreoretinal interface can induce Müller glia gliosis and are implicated in epiretinal membrane (ERM) formation [16] and retinal detachment [3, 17]. Similarly, elevated shear stress at the inner BRB compromises its integrity [12], provoking Müller glia‐mediated inflammatory responses at the *glia limitans* (a barrier formed by Müller glia end‐feet around the BRB). Additionally, shear forces generated by subretinal fluid accumulation may also disrupt Müller glia anchoring, promoting tissue separation and contributing to retinal detachment [18].

Despite these observations, the direct effects of shear stress on Müller glia remain largely unexplored. Although shear stress within the retina has been estimated to be on the order of 0.1 dyn/cm^2^ [19], these values are derived primarily from computational modelling of tractional stresses at the vitreoretinal interface, often under harsh pathological conditions such as head trauma. Importantly, such models do not provide direct measurements of laminar shear stress acting on Müller glia; to our knowledge, no studies have directly assessed Müller glial responses to such forces. This knowledge gap is particularly relevant considering their strategic positioning at the interface of neural, vascular, and fluid compartments, as well as their established responsiveness to other mechanical stimuli. Notably, other glial populations within the central nervous system (CNS), such as radial glia, astrocytes and microglia, respond to shear stress magnitudes in the range of 10^−3^–10^−2^ dyn/cm^2^ by modulating cytoskeletal architecture and gene expression profiles [20, 21, 22, 23]. These findings strongly suggest that Müller glia may also possess a high degree of mechanosensitivity to physiological or pathological shear forces. Investigating how shear stress influences Müller glia is thus essential to fully understand their role in retinal homoeostasis, neurovascular interactions and the initiation or progression of mechanopathologies such as fibrosis and detachment.

To address this gap, primary adult Müller glia cultures provide a robust model for studying mechanobiological responses under controlled conditions. Building on our previous work with adult Müller glia cultures [24] and utilising a custom‐designed microfluidic system developed by the van Oterendorp Lab [25, 26], we applied fluid flow to induce shear stress *in vitro*. Therefore, this study aims to characterise how shear stress influences key aspects of Müller glia physiology, including cell survival, morphology and ECM deposition, and to identify the contribution of TRPV4 and TGF‐β1 pathways in these processes. Ultimately, dissecting how Müller glia interpret and respond to fluid flow‐induced mechanical cues could reveal novel therapeutic avenues for preventing or mitigating tractional pathologies, such as ERM formation and retinal detachment.

## 2 Materials and Methods

### 2.1 Animals

Adult 4‐month‐old Wistar rat eyes (*n* = 18) were obtained from animals reared at the Universitätsmedizin Göttingen animal house. Animals were kept in standard housing conditions on a 12 h light–dark cycle, with *ad libitum* access to food and water. Rats were humanely sacrificed by exposure to CO_2_. The experimental protocol met European (2010/63/UE) standards for the protection of experimental animals, and it was approved by the Ethical Committee for Animal Welfare of the Universitätsmedizin Göttingen.

### 2.2 Isolation and Culture of Adult Müller Glia

Immediately after enucleation, rat eyes were dissected to isolate the retinas using fresh CO₂‐independent Dulbecco's Modified Eagle's Medium (DMEM/CO_2_; Gibco‐Life Technologies, Waltham, MA, United States).

Müller glia cultures were obtained as described by Pereiro et al. [24]. The retinas were incubated at 37°C for 30 min in a Sterile Earle's Balanced Salt Solution containing 20 U/mL Papain and 2000 U/mL DNase (Worthington, Lakewood, NJ, United States). Enzymatic digestion was halted by adding DMEM supplemented with 10% foetal bovine serum (FBS) for 3 min at room temperature. The tissue was then mechanically dissociated using pipettes with decreasing tip diameters, and the resulting cell suspension was centrifuged at 1200 rpm for 5 min.

Pelleted cells were re‐suspended in DMEM + 10% FBS medium and seeded on single‐channel µ slides I, Luer (0.8 mm height, 2.5 cm^2^ growth area; Ibidi, Gräfelfing, Germany) at a density of 2.5 × 10^6^ cells per channel. Prior to seeding, flow channels were coated overnight with 100 μg/mL poly‐L‐lysine (Sigma‐Aldrich, St. Louis, MO, United States) in distilled water, followed by incubation with 10 μg/mL laminin (Sigma‐Aldrich, St. Louis, MO, United States) in Phosphate Buffer Saline (PBS; Gibco‐Life Technologies, Waltham, MA, United States) for at least 2 h. Cultures were maintained in a humidified incubator at 37°C in an atmosphere of 5% CO_2_ and 95% O_2_. The culture medium was fully replaced on 1 day *in vitro* (DIV). Subsequently, half of the medium was changed every 2 days.

### 2.3 Cultures Exposed to Shear Stress

To test the response of Müller glia to shear stress, cells were seeded in single‐channel slides. After 6DIV, cells were subjected to a 10^−3^ dyn/cm^2^ flow for 24 h before fixing. This 6 DIV + 24 h schedule was chosen during model optimisation: 2–4 DIV did not achieve confluence, whereas by 8 DIV (9 total days including flow), cells showed declining health.

Likewise, the shear stress magnitude was selected during the initial establishment of the model. Multiple magnitudes were tested: moderate shear stress (10^−3^ dyn/cm^2^) consistently supported cell survival and maintained characteristic morphology, higher shear stress (10^−2^ dyn/cm^2^) markedly reduced viability, precluding further analysis and lower shear stress (10^−6^ dyn/cm^2^) elicited similar but less consistent responses (Figure S1). This value was also supported by previous studies on other glial cells [23]. Flow was achieved by injecting fresh medium at a constant rate of 3.793 µL/min using a Pump33DDS system (Harvard Apparatus, Holliston, MA, United States) connected to a standard incubator to maintain temperature, air and humidity conditions.

### 2.4 TRPV4 Inhibition

To evaluate the role of the mechanosensitive channel TRPV4 in the response of Müller glia to shear stress, cells were treated with 10 μM of TRPV4 inhibitor HC‐067472 (Merck‐Millipore, Burlington, MA, United States) [27] before subjecting them to flow conditions for 24 h. Control cultures were also treated with 10 μM of HC‐067472 and kept at static conditions in a standard cell incubator.

### 2.5 TGF‐β1 Inhibition

To determine the effect of TGF‐β1 on Müller glia response to flow‐induced shear stress, cells were treated with 10 μg/mL of TGF‐β1 inhibitor SB‐431542 (CaymanChemical, Ann Arbor, MI, United States) [28]. Cells were then subjected to flow for 24 h. Control cultures were also treated with 10 μg/mL of SB‐431542 and kept at static conditions in a standard cell incubator.

### 2.6 Immunocytochemistry

After 7DIV, cells were washed in PBS, fixed in methanol for 10 min at −20°C and nonspecific antigen binding was blocked for 30 min at room temperature with blocking buffer (0.25% Triton X‐100 and 3% bovine serum albumin in PBS). The primary antibodies (see Table 1) were diluted in blocking buffer and incubated with the cultures overnight at 4°C. The cells were washed three times with PBS and the cultures were exposed for 1 h at room temperature to the corresponding secondary antibodies at a dilution of 1:1000 in blocking buffer: Alexa Fluor 488 and Alexa 555 conjugated goat anti‐mouse and goat anti‐rabbit antibodies (Invitrogen, Eugene, OR, United States) and counterstained with 4’,6‐diamidino‐2‐phenylindole (DAPI; Invitrogen, Eugene, OR, United States) at a dilution of 1:10000. After three washes, the cultures were kept in PBS.

**Table 1:** List of primary antibodies.

Antigen	Target	Host	Dilution	Supplier
Collagen I (col1a2)	ECM	Rabbit	1:500	Sigma
Collagen IV (col4a1)	ECM	Rabbit	1:500	Sigma
Fibronectin	ECM	Rabbit	1:500	Sigma
pFAK	Focal adhesion	Rabbit	1:200	ThermoFisher
TGF‐β1	Fibrosis	Rabbit	1:500	Abcam
TRPV4	Mechanosensor	Rabbit	1:500	LS‐Bio
Vimentin	Müller glia	Mouse	1:1000	Dako

### 2.7 TUNEL Assay

To assess whether alterations in cell number and cell area were due to an adaptive response of Müller glia or to cell death, we performed a terminal deoxynucleotidyl transferase dUTP nick end labelling (TUNEL) assay. Briefly, after cells were fixed and immunostained, they were incubated with the TUNEL reaction mixture containing 45 μL TUNEL Label Mix and 5 μL of TUNEL Enzyme (Roche, Basel, Switzerland). After 1 h in incubation at 37°C, samples were washed thrice and kept in PBS. To confirm the assay's functionality, Müller glia were subjected to hypoxia as a positive control.

### 2.8 Image Acquisition and Processing

Müller glia were analysed on an epifluorescence microscope (Zeiss, Jena, Germany) coupled to a digital camera (Zeiss Axiocam MRM, Zeiss, Jena, Germany). Image acquisition was controlled by ZENpro software (Zeiss, Jena, Germany). All images were taken with the same settings (i.e., exposure time, aperture and lighting intensity).

To evaluate cell survival under each condition, Müller glia were manually counted using ImageJ software (1.48 v; NIH, Bethesda, MD, USA). Cells were considered surviving when adherent cell bodies with intact morphology and nuclei were present within the imaging field, allowing exclusion of detached material or debris. Cell morphology was analysed by manually tracing the cell contours with the Freehand Selection tool in ImageJ, after which the cell area was automatically calculated.

The expression levels of TRPV4 and TGF‐β1 were quantified as the integrated fluorescence density within the cells, using ImageJ [29]. Similarly, fluorescence intensity corresponding to ECM deposition was measured, with the integrated density calculated after subtracting the fluorescence signal expressed by cells themselves. All integrated density values were normalised to the number of surviving cells in each condition to account for treatment‐dependent differences in cell density.

To quantify focal adhesion‐associated phosphorylated focal adhesion kinase (pFAK), fluorescence intensity was measured at the level of discrete puncta using ImageJ. pFAK‐positive puncta were identified based on their characteristic punctate morphology. Line scans were manually drawn across pFAK‐positive puncta using the straight‐line tool, ensuring that the line spanned both the puncta and adjacent background regions. Fluorescence intensity profiles were obtained. From these profiles, peak intensity values were extracted relative to the background.

### 2.9 Statistical Analysis

Experimental procedures were replicated at least three times for each experiment, and at least 30 photos were taken for each condition. Results were normalised to control conditions: static, no TGF‐β1/TRPV4 inhibition. For pFAK analysis, at least 20 puncta were analysed per cell. For each independent experiment, a minimum of 10 cells per condition were analysed. All measurements were performed using identical imaging and analysis parameters to ensure consistency.

Statistical analyses were carried out with SPSS Statistical software (24‐0 v; IBM, Armonk, NY, United States), and the means and standard error of the mean (SEM) for each condition are presented. A Kruskal–Wallis nonparametric test, followed by a post hoc Dunn test, was used to evaluate whether there were significant differences between means. Differences were considered significant for all tests at *p*‐value ≤ 0.05.

## 3 Results

### 3.1 Müller Glia Survival Is Enhanced and Cell Area Reduced Under Flow‐Induced Shear Stress

**Figure 1:**
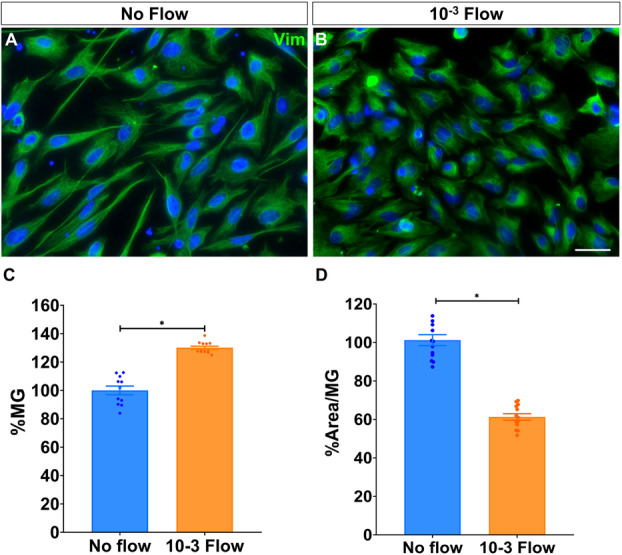
Müller glia survival is enhanced and cell area reduced under flow‐induced shear stress. Images of Müller glia cultured (A) under static conditions or (B) after being under a 10^−3^ dyn/cm^2^ flow for 24 h. (C, D) Histograms of the percentage of Müller glia and their cell area in the cultures under flow relative to the control. Müller glia (green) were labelled with an antibody against vimentin. Nuclei (blue) were stained with DAPI. Data are represented as mean ± SEM. *p‐value < 0.05. Scale bar: 50 μm.

Given Müller glia involvement in mechanosensing, we assessed their response to flow‐induced shear stress, a mechanical cue essential for retinal vascular homoeostasis and relevant to outcomes following retinal detachment. To this end, Müller glia were cultured in single‐channel flow chambers and exposed to a shear stress of 10^−3^ dyn/cm^2^ for 24 h (Figure 1).

Notably, shear stress significantly enhanced the number of Müller glia (130.12% ± 1.07%) compared to static or no flow (control) conditions (100.00% ± 3.64%; Figure 1C). Interestingly, although cells displayed a fusiform morphology in both conditions, shear stress reduced Müller glia cell area (61.29% ± 1.63%; Figure 1D). These differences appear to be an adaptive response to flow‐induced shear stress, rather than evidence of cell death, as suggested by the TUNEL assay (see Figure S2A,B).

### 3.2 TRPV4 Mediates Müller Glia Survival and Morphology Changes in Response to Shear Stress

**Figure 2:**
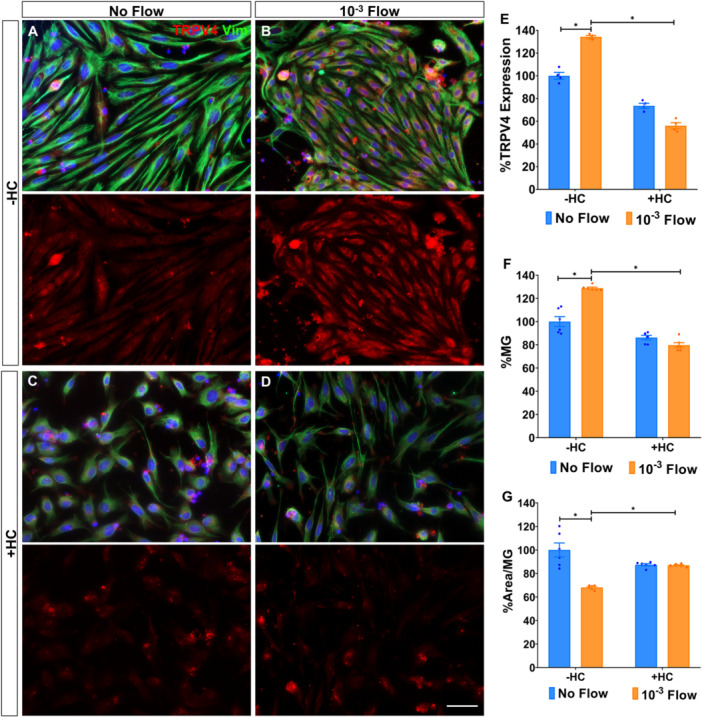
TRPV4 mediates Müller glia survival and morphology changes in response to shear stress. Expression of the mechanosensitive channel TRPV4 by Müller glia cultured (A) under static conditions or (B) after being subjected to a 10^−3^ dyn/cm^2^ flow for 24 h. (C, D) Cells in both conditions were also treated with 10 μM of the TRPV4 inhibitor HC‐067472 (HC). The same number of cells was seeded on all conditions. (E) The percentage of TRPV4 expressed by Müller glia relative to untreated static cultures. (F, G) Histograms of the percentage of Müller glia and their cell area in the cultures under flow relative to the control. Müller glia were labelled with antibodies against vimentin (green) and TRPV4 (red). Nuclei (blue) were stained with DAPI. Data are represented as mean ± SEM. *p‐value < 0.05. Scale bar: 50 μm.

As TRPV4 channel activation is susceptible to different mechanical stresses, we analysed TRPV4 expression (Figure 2). Müller glia expressed significantly higher levels of TRPV4 when subjected to flow (134.31% ± 1.16%; Figure 2B). To further confirm the mechanosensitive role of TRPV4 in shear stress sensing, we pharmacologically inhibited the channel using HC‐067472 (HC) for 24 h. While HC‐067472 treatment had no significant effect on TRPV4 expression in static cultures, it substantially reduced TRPV4 signalling under flow conditions (56.13% ± 2.65%; Figure 2D).

Notably, TRPV4 inhibition under flow decreased Müller glia survival (79.71% ± 2.12% vs. 128.84% ± 0.88%) and increased cell area (86.90% ± 0.50% vs. 68.10% ± 0.50%), without significantly affecting cells under static conditions (Figure 2C).

### 3.3 Flow‐Induced pFAK Signalling in Müller Glia Is Mediated by TRPV4 Activation

**Figure 3:**
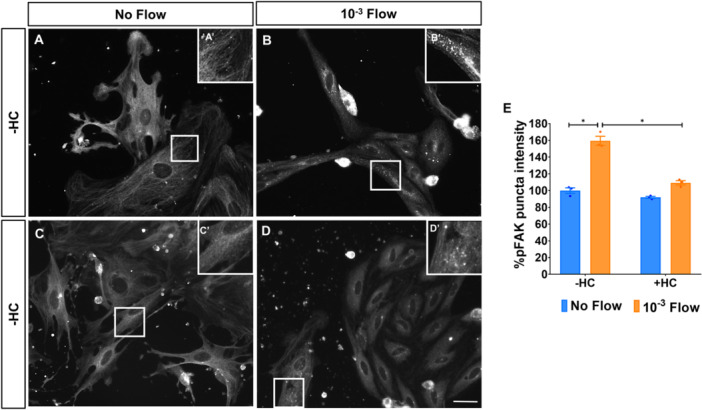
Flow‐induced pFAK signalling in Müller glia is mediated by TRPV4 activation. Expression of the cell‐matrix adhesion protein pFAK by Müller glia cultured (A) under static conditions or (B) after being subjected to a 10^−3^ dyn/cm^2^ flow for 24 h. (C, D) Cells in both conditions were also treated with 10 μM of the TRPV4 inhibitor HC‐067472 (HC). (A’–D’) High‐magnification insets showing pFAK organisation. The same number of cells was seeded on all conditions. (E) Intensity of pFAK puncta expressed by Müller glia relative to untreated static cultures. Müller glia were labelled with antibody against pFAK. Data are represented as mean ± SEM. *p‐value < 0.05. Scale bar: 50 μm.

Since TRPV4 has been linked to cytoskeletal contraction in response to flow, we also assessed the expression of pFAK in Müller glia, a protein involved in cell–matrix interactions and recruited during this process. In alignment with TRPV4 activation, Müller glia exposed to flow exhibited significantly higher levels of pFAK (159.49% ± 5.39%; Figure 3B). Furthermore, pFAK was organised as distinct puncta under flow, while under static conditions appeared more diffuse (Figure 3A’, B’).

HC‐067472 treatment did not significantly affect pFAK intensity levels nor organisation under static conditions (Figure 3C,C’) but did reduce flow‐induced pFAK intensity to values closer to control conditions (109.30% ± 2.54%; Figure 3D). Interestingly, although discrete puncta could still be observed under flow, the general pattern was more similar to static conditions (Figure 3D’). These results suggest that TRPV4 activation contributes to flow‐induced pFAK signalling and organisation in Müller glia.

### 3.4 Shear Stress‐Induced TGF‐β1 Signalling Promotes Müller Glia Survival and Influences Cell Morphology

**Figure 4:**
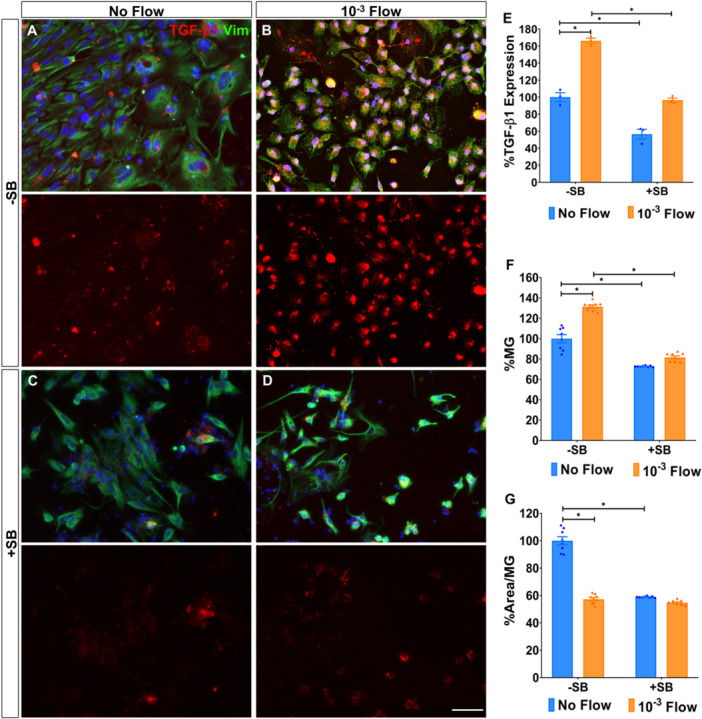
Shear stress‐induced TGF‐β1 signalling promotes Müller glia survival and influences cell morphology. Expression of TGF‐β1 by Müller glia cultured (A) under static or (B) 10^−3^ dyn/cm^2^ flow conditions. (C, D) Cells in both conditions were treated with 10 μg/mL of the TGF‐β1 inhibitor SB‐431542 (SB). The same number of cells was seeded on all conditions. (E) The percentage of TGF‐β1 expressed by Müller glia relative to untreated static cultures. (F, G) Histograms of the percentage of Müller glia and their cell area in the cultures under flow relative to the control. Müller glia were labelled with antibodies against vimentin (green) and TGF‐β1 (red). Nuclei (blue) were stained with DAPI. Data are represented as mean ± SEM. *p‐value < 0.05. Scale bar: 50 μm.

Having established that shear stress activates TRPV4 and cell‐matrix pFAK in Müller glia, we next evaluated whether it also triggered TGF‐β1 signalling. Indeed, TGF‐β1 expression significantly increased in response to flow (166.21% ± 3.09%; Figure 4B). To further assess the functional role of TGF‐β1, we treated cells with the inhibitor SB‐431542 for 24 h, which effectively suppressed TGF‐β1 expression under both static (56.53% ± 5.65%) and flow conditions (96.52% ± 2.42%; Figure 4C,D).

TGF‐β1 inhibition also reduced Müller glia survival in both static (72.84% ± 0.24% vs. 100.00% ± 3.92%) and flow (81.59% ± 1.58% vs. 131.50% ± 2.25%) conditions, while SB‐431542 treatment only affected cell area under static conditions (58.95% ± 0.17% vs. 100.00% ± 2.75%; Figure 4A,C). Nonetheless, SB‐431542 treatment did not affect the apoptotic state of Müller glia (Figure S2C,D).

### 3.5 Shear Stress Induces TGF‐β1–Dependent ECM Remodelling in Müller Glia Without Collagen Deposition

Given the upregulation of TGF‐β1 by shear stress, we next investigated ECM remodelling under flow.

**Figure 5:**
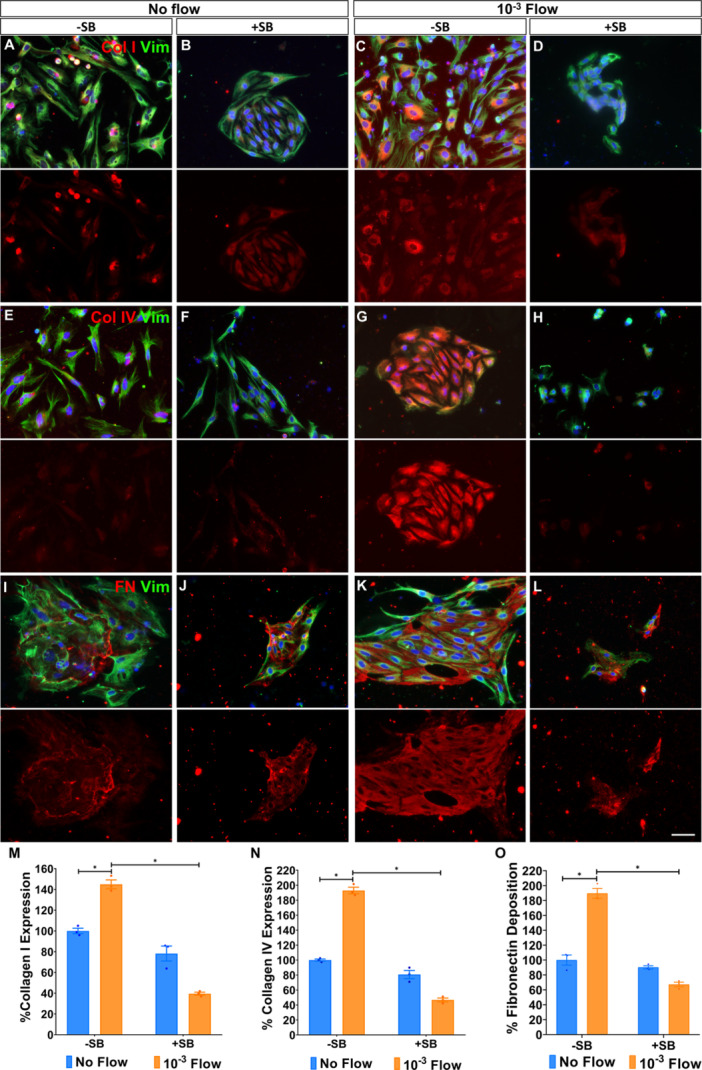
Shear stress induces TGF‐β1‐dependent ECM remodelling in Müller glia without collagen deposition. Müller glia expression of (A–D) collagen I, (E–H) collagen IV and (I–L) fibronectin under (A–B, E–F, I–J) static or (C–D, G–H, K–L) a 10^−3^ dyn/cm^2^ flow conditions. (B, D, F, H, J, K) Cells in both conditions were also treated with 10 μg/mL of the TGF‐β1 inhibitor SB‐431542 (SB). The same number of cells was seeded on all conditions. Histograms of the percentage of Müller glia expression of (M) collagen I and (N) collagen IV, and (O) fibronectin deposition, and their cell area in the cultures under flow relative to untreated static cultures. Müller glia were labelled with antibodies against vimentin (green), collagen I (red, A–D) collagen IV (red, E–H) and fibronectin (red, I–L). Nuclei (blue) were stained with DAPI. Data are represented as mean ± SEM. *p‐value < 0.05. Scale bar: 50 μm.

Surprisingly, while intracellular levels of collagen I and IV increased under flow (144.93% ± 4.26% and 193.05% ± 4.38%, respectively; Figure 5C,G), neither was deposited extracellularly. Nonetheless, this expression was also TGF‐β1‐dependent, as SB‐431542 treatment markedly reduced collagen I (39.51% ± 1.46%) and collagen IV (46.73% ± 2.59%) levels under flow (Figure 5D,H).

In contrast, Müller glia did deposit fibronectin, which significantly increased under flow (189.52% ± 6.60%; Figure 5K), and inhibition of TGF‐β1 significantly reduced its deposition (67.27% ± 3.09%; Figure 5L).

## 4 Discussion

Mechanical cues are emerging as critical regulators of retinal glial physiology, yet their specific roles remain incompletely understood [1]. While prior studies of our group have shown that Müller glia respond to substrate stiffness and hydrostatic pressure by altering their phenotype and ECM production [10], the specific impact of fluid shear stress has remained largely uncharacterised. Given that shear stress arises from physiological processes, such as intraocular fluid flow and BRB dynamics [11, 12], and is exacerbated under pathological conditions like retinal detachment [18] or ERM formation [16], understanding how Müller glia respond to this stimulus is essential for deciphering the mechanobiology of retinal disease. To evaluate this response, Müller glia were cultured on flow single‐channel slides and subjected to a 10^−3^ dyn/cm^2^ flow for 24 h. This shear range was selected based on prior studies showing responsiveness in other CNS glial cells (i.e., astrocytes, microglia, cortical radial glial cells) [20, 21, 22, 23]. These reports suggested that Müller glia would similarly exhibit mechanosensitive behaviour under flow.

We first observed that shear stress enhanced Müller glia survival. While the effects of shear stress on Müller glia remain poorly characterised, recent transcriptomic analyses from a diabetic retinopathy model showed enrichment of pathways linked to cell survival under fluid shear stress sensing [30]. This is consistent with findings in other glial populations, such as astrocytes, cortical radial glial cells and microglia, where moderate levels of shear stress promote survival, proliferation, and activation [22, 23, 31], suggesting a conserved survival‐promoting effect across glial populations in the CNS.

Interestingly, despite the observed increase in survival, Müller glia subjected to shear stress exhibited a significant reduction in cell area. This morphological adaptation contrasts with most reports in other glial cells, where shear typically induces cell spreading or hypertrophy. In Müller glia, however, this response may reflect a contraction‐like phenotype driven by cytoskeletal remodelling, potentially aimed at minimising surface exposure and enhancing resistance to mechanical deformation. Such a response is consistent with Müller glia's response to BRB disruption, retinal tear/detachment or ERM formation, events that generate shear stress and where Müller glia proliferate and participate in fibrocellular contraction processes [32]. A similar behaviour has been reported in other cell types, such as vascular endothelial cells and fibroblasts, as a protective mechanism [33, 34]. Thus, the reduction in Müller glia cell area may reflect a specialised response that aligns more closely with barrier‐forming or structural support cells than with motile glia such as astrocytes or microglia.

Given these shear‐induced morphological changes and their potential link to cytoskeletal tension, we examined the role of the mechanosensitive calcium‐channel TRPV4, which has been shown to trigger actomyosin contractility in response to mechanical cues [35]. Previous reports show that shear stress is able to activate TRPV4 channels [36, 37]. Previous studies have demonstrated TRPV4 activation by shear stress in various cell types, including vascular cells [38], as well as astrocytes [20]. Consistent with this, shear stress robustly upregulated TRPV4 expression in Müller glia, supporting its role as a mechanotransducer in response to flow [6].

Pharmacological inhibition of TRPV4 with the selective antagonist HC‐067472 (HC) [27] significantly attenuated TRPV4 signalling, impaired Müller glia survival under flow, without affecting cells under static conditions. This context‐dependent reduction in viability suggests that TRPV4 activity is specifically required for the adaptive survival response to shear stress, highlighting TRPV4 as a key mediator of mechanotransductive survival signalling in Müller glia. This interpretation is consistent with previous reports in endothelial and epithelial cells, where TRPV4‐mediated calcium influx under flow activates downstream survival pathways [37, 39].

In addition to its impact on survival, TRPV4 inhibition also partially reversed the reduction in cell area observed under flow, suggesting a direct role for this channel in cytoskeletal remodelling. TRPV4 activation has been linked to actomyosin contractility via calcium‐dependent RhoA/ROCK signalling, a pathway known to regulate morphological responses such as cell area reduction and increased cortical tension, as observed in the trabecular meshwork [40]. These pathways have also been shown to influence Müller glia viability and ECM production [41]. To further explore this mechanism, we assessed the levels of pFAK, a key effector of RhoA/ROCK signalling involved in many processes, including cell proliferation, migration, differentiation and ECM production [42, 43] and expressed by Müller glia [44]. Consistent with these reports, Müller glia levels of pFAK significantly increased under flow, and presented as puncta, and were markedly reduced by TRPV4 inhibition, confirming the role of TRPV4 in shear stress‐mediated cytoskeletal remodelling. The punctate intracellular organisation of pFAK under flow is consistent with mechanosensitive clustering of adhesion signalling complexes associated with cytoskeletal tension and actomyosin contractility, a spatial reorganisation that has been linked to force transmission and adaptive remodelling in mechanically stimulated cells [45]. Moreover, similar adaptive contractile responses have been observed in Müller glia under other forms of mechanical stress, such as low substrate stiffness [10], supporting the idea that this reduction in cell area represents a mechanoadaptive strategy.

Given the well‐established role of TRPV4 in activating TGF‐β1 signalling [9], we also explored the contribution of this pro‐fibrotic cytokine in Müller glia shear stress‐induced responses. Shear stress led to a significant upregulation of TGF‐β1 expression in Müller glia, consistent with previous with prior studies in vascular and epithelial cells [46, 47], suggesting that TGF‐β1 may play a role in mediating ECM remodelling under shear stress conditions. Inhibition of TGF‐β1 signalling using its selective antagonist SB‐431542 (SB) [28] not only suppressed its expression but also significantly reduced Müller glia survival under both static and flow conditions. This reduction supports a broader role for TGF‐β1 signalling in maintaining Müller glial [10] and other retinal cells' viability [48]. Under flow, inhibition likely disrupts survival pathways that contribute to cellular adaptation, reinforcing the interpretation that TGF‐β1 is an integral component of the mechanosensitive survival response.

Despite its influence on survival, TGF‐β1 inhibition did not affect Müller glia morphology under flow, although it significantly reduced cell area under static conditions. This finding suggests that, under flow, morphological adaptation is primarily driven by TRPV4‐dependent mechanisms (i.e., actomyosin contractility via its effector pFAK) [49]. This interpretation is consistent with reports showing that shear stress can activate the RhoA/ROCK pathway independently of TGF‐β1 [50], supporting the notion that TRPV4–calcium–RhoA/ROCK signalling may bypass the requirement for TGF‐β1 in regulating cell morphology under flow.

We further examined whether TGF‐β1 activation leads to ECM remodelling of key components, collagen I, collagen IV and fibronectin. Remarkably, we found a striking dissociation between collagen I and collagen IV expression and their deposition under shear stress conditions. Specifically, while collagen I and IV expression increased significantly within Müller glia subjected to flow, extracellular deposition of these proteins was nearly absent. Nevertheless, this expression was sensitive to TGF‐β1 inhibition, indicating that TGF‐β1 drives collagen gene expression, even though its deposition appears decoupled. Previous studies have shown that low shear stress enhances collagen organisation in chondrocytes and corneal fibroblasts, whereas higher shear can inhibit proper collagen assembly [51, 52]. Similarly, collagen deposition in astrocytes and glioblastoma cells has been reported to depend on specific thresholds and durations of mechanical input [53, 54, 55]. For Müller glia, although the shear stress induced in our experiments was enough to activate the transcription of collagen I and IV, these cells may require a higher shear stimulus or longer exposure for the maturation and deposition of collagen into the extracellular space [56, 57, 58]. Alternatively, secreted collagen could be flushed out with the medium before it can assemble.

Unlike collagen, fibronectin was deposited under both static and flow conditions. Moreover, this deposition was significantly increased when Müller glia were subjected to shear stress and SB‐431542 treatment markedly reduced fibronectin deposition, supporting a clear role for TGF‐β1 in fibronectin assembly. This differential ECM response, where fibronectin is deposited despite the absence of collagen, highlights the distinct roles that fibronectin and collagen play in ECM organisation. Fibronectin, as an early‐stage ECM component, forms a provisional matrix that supports cell adhesion and migration and is less dependent on mechanical tension than collagen [59, 60, 61]. Fibronectin is deposited and reorganised more readily than collagen, even under low mechanical tension [62], and may serve as a provisional matrix for subsequent, more structured collagen deposition. Our findings are consistent with studies showing that fibronectin can be rapidly assembled into a matrix by cells exposed to fluid shear stress [63, 64], and support a role for Müller glia in the early phases of ECM remodelling in response to injury‐associated flow, such as in BRB disruption.

## 5 Conclusions

In this study, we demonstrate that Müller glia are mechanosensitive cells capable of integrating shear stress signals to orchestrate distinct morphological, survival, and fibrotic responses *in vitro*. Exposure to shear stress enhances Müller glia survival, induces a reduction in cell area, and promotes selective ECM remodelling, highlighting their capacity to adapt to dynamic mechanical environments. Our findings identify TRPV4 as a central mechanosensor that mediates both the survival and morphological responses of Müller glia under shear stress, partially through actomyosin contractility and pFAK activation. Moreover, our data reveal that while TGF‐β1 supports fibronectin deposition and collagen expression, extracellular collagen assembly appears limited under the shear conditions tested. This decoupling suggests that additional mechanical or temporal cues may be required for full ECM maturation and fibrotic remodelling and highlights Müller glia as initiators of matrix remodelling in early or sub‐threshold mechanical injury. Importantly, pharmacological inhibition of TRPV4 or TGF‐β1 abrogates these responses, underscoring their functional relevance in Müller glia mechanobiology.

These findings have direct implications for retinal pathologies characterised by abnormal mechanical forces and glial activation, such as retinal detachment, BRB breakdown and ERM formation. In these contexts, Müller glial mechanotransduction may act as an upstream driver of neuroinflammation, gliosis, and fibrotic scarring. Targeting mechanosensitive pathways such as TRPV4 and TGF‐β1 signalling could therefore offer novel therapeutic avenues to preserve retinal architecture, limit glial reactivity, and prevent vision‐threatening fibrosis.

## Author Contributions

Conceptualisation: Xandra Pereiro and Elena Vecino. Methodology: Laura Prieto‐López. Validation: Elena Vecino and Xandra Pereiro; Formal analysis and Investigation: Laura Prieto‐López. Resources: Christian van Oterendorp and Elena Vecino. Data curation and Writing–original draft preparation: Laura Prieto‐López. Writing–review and editing: Christian van Oterendorp, Elena Vecino and Xandra Pereiro. Visualisation: Laura Prieto‐López and Xandra Pereiro. Supervision: Christian van Oterendorp, Elena Vecino and Xandra Pereiro. Project administration: Elena Vecino. Funding acquisition: Elena Vecino. All authors have read and agreed to the published version of the manuscript.

## Conflicts of Interest

The authors declare no conflicts of interest.

## Supporting information

Supporting File 1:

Supporting File 2:

## Data Availability

Data will be provided on demand.
