# Supplementary figures and images for: Shear Stress as a Driver of Retinal Müller Glia Survival and Fibrotic Reprogramming
Source: Cell Biochem Funct. 2026 May 11;44:e70224. doi: 10.1002/cbf.70224 (PMC13161832; doi:10.1002/cbf.70224)

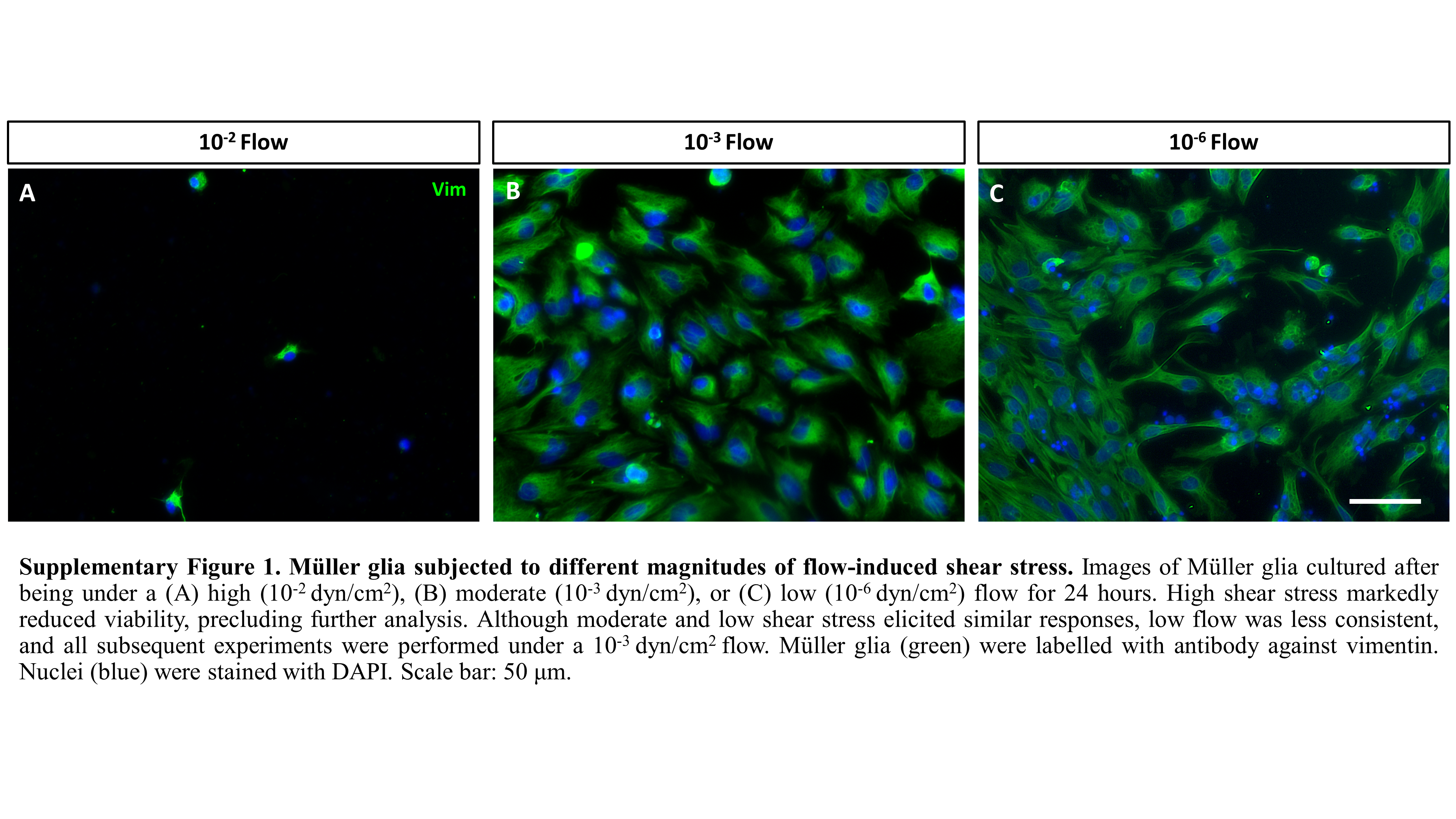

Supplement: Supplementary file 1 — Supporting File 1: [file CBF-44-e70224-s002.tif]

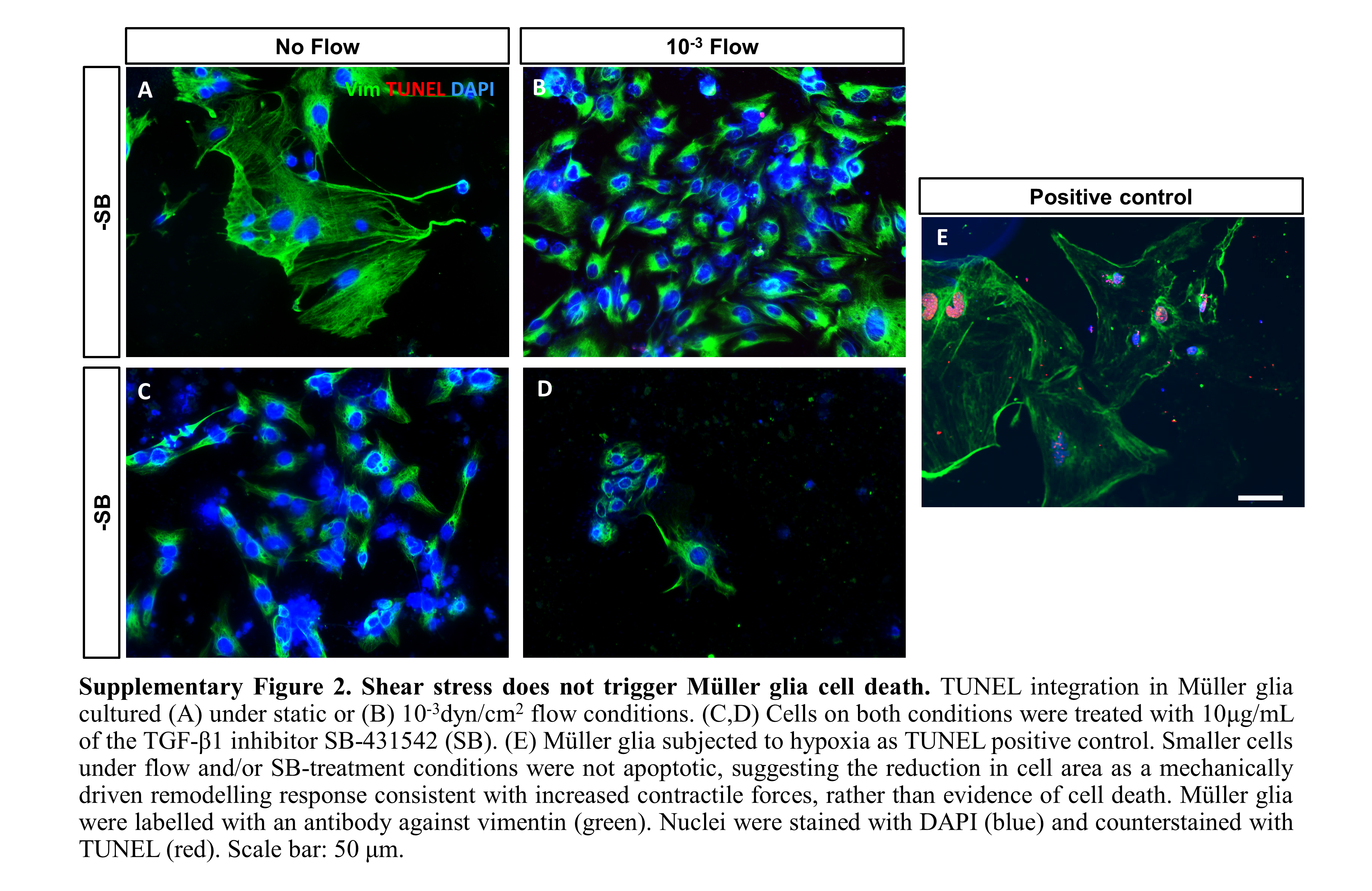

Supplement: Supplementary file 2 — Supporting File 2: [file CBF-44-e70224-s001.tif]
